# Incidence, prevalence, and national burden of interstitial lung diseases in India: Estimates from two studies of 3089 subjects

**DOI:** 10.1371/journal.pone.0271665

**Published:** 2022-07-21

**Authors:** Sahajal Dhooria, Inderpaul Singh Sehgal, Ritesh Agarwal, Valliappan Muthu, Kuruswamy Thurai Prasad, Soundappan Kathirvel, Mandeep Garg, Amanjit Bal, Ashutosh Nath Aggarwal, Digambar Behera

**Affiliations:** 1 Department of Pulmonary Medicine, Postgraduate Institute of Medical Education and Research (PGIMER), Chandigarh, India; 2 Department of Community Medicine and School of Public Health, Postgraduate Institute of Medical Education and Research (PGIMER), Chandigarh, India; 3 Department of Radiodiagnosis and Imaging, Postgraduate Institute of Medical Education and Research (PGIMER), Chandigarh, India; 4 Department of Histopathology, Postgraduate Institute of Medical Education and Research (PGIMER), Chandigarh, India; Al-Azhar University, EGYPT

## Abstract

**Background and objective:**

The epidemiology of interstitial lung diseases (ILDs) in developing countries remains unknown. The objective of this study was to estimate the incidence, prevalence, and national burden of ILDs in India.

**Methods:**

Data of consecutive subjects (aged >12 years) with ILDs included in a registry between March 2015 and February 2020 were analyzed retrospectively. The proportion of each ILD subtype was determined. The crude annual incidence and prevalence of ILDs for our region were estimated. Subsequently, the primary estimates of the national annual incident and prevalent burden of ILD and its subtypes were calculated. Alternative estimates for each ILD subtype were calculated using the current and a large, previous Indian study (n = 1,084). Data were analyzed using SPSS version 22 and are presented descriptively.

**Results:**

A total of 2,005 subjects (mean age, 50.7 years; 47% men) were enrolled. Sarcoidosis (37.3%) was the most common ILD subtype followed by connective tissue disease (CTD)-related ILDs (19.3%), idiopathic pulmonary fibrosis (IPF, 17.0%), and hypersensitivity pneumonitis (HP, 14.4%). The crude annual incidence and prevalence of ILDs were 10.1–20.2 and 49.0–98.1, respectively per 100,000 population. The best primary estimates for the crude national burden of all ILDs, sarcoidosis, CTD-ILD, IPF, HP, and other ILDs (in thousands) were 433–867, 213–427, 75–150, 51–102, 54–109, and 39–78. The respective alternative estimates (in thousands) were sarcoidosis, 127–254; CTD-ILD, 81–162; IPF, 46–91; HP, 130–261; other ILDs, 49–98.

**Conclusion:**

In contrast to developed countries, sarcoidosis and HP are the ILDs with the highest burden in India.

## Introduction

Interstitial lung diseases (ILDs) are disorders characterized by non-infective inflammation or fibrosis diffusely affecting the lung parenchyma [1]. The major subtypes include sarcoidosis, idiopathic pulmonary fibrosis (IPF), connective tissue disease-related ILD (CTD-ILD), hypersensitivity pneumonitis (HP), and others [2, 3].

The crude annual incidence of ILDs ranges from 1 to 70.1 per 100,000 population in different studies worldwide [4–16] while the prevalence lies between 6.27 and 97.9 per 100,000 population [4, 5, 10, 13]. Most previous studies have not used the contemporary classification proposed by the latest American Thoracic Society/European Respiratory Society consensus statements [1, 2]. Also, no study has reported the incidence and prevalence of ILDs from developing countries. In the developing world, non-communicable respiratory diseases remain underrecognized due to the high burden of infectious diseases such as tuberculosis [17]. There is an unmet need for epidemiologic data on ILDs from India, the world’s second most populous country. Such knowledge can better inform national and international efforts for patient care and research in ILDs.

The spectrum of ILD subtypes at our center has been previously described [3]. Herein, we describe the incidence and prevalence of ILDs in our region located in northern India. The national incident and prevalent burdens of ILD and its subtypes have also been estimated using the current study and a previous large multicenter study from India [18, 19].

## Methods

In this study, data of subjects enrolled into an ILD registry at our Chest Clinic between March 2015 and February 2020 were analyzed retrospectively. The study protocol (Pulm653) was approved by the Institutional Ethics Committee, Postgraduate Institute of Medical Education and Research, Chandigarh, India. Written informed consent was obtained from all the subjects for participation in the registry. Consent was obtained from parents or guardians for the minors included in the study. We have previously published the data of a part of the study population included in the current study [3].

### Subjects and study procedures

Subjects were enrolled into our ILD registry if they met all the following criteria: (i) age >12 years (adolescents and adults); (ii) diagnosis of ILD; and, (iii) willingness to provide informed consent. Subjects with any of the following were excluded: (i) final diagnosis of a disease other than an ILD; and (ii) lack of informed consent. The demographic details, spirometric measurements, the final diagnosis, and the dates of diagnosis and death were extracted from the registry data. The proportion of each ILD subtype was calculated.

### Diagnosis of ILD and its subtypes

In our Chest Clinic, all subjects with a suspected ILD were referred to one author (SD) for inclusion into the ILD registry. A detailed history was obtained, including the symptoms, the risk factors for various ILDs, family history, history of exposures to cigarette smoke, drugs, other environmental dusts, and the presence of any connective tissue disease (CTD). A thin section (0.5–1.5 mm) computed tomography (CT) of the chest, spirometry, and serology for autoimmune diseases were obtained; further tests were guide by the suspected diagnosis. Lung biopsy or other invasive procedures were performed for obtaining tissue samples if indicated [20, 21]. The diagnosis of the ILD subtype was made as described previously [3] using contemporary guidelines, statements, or expert opinions [1, 2, 22–27]. In general, subjects with suspected sarcoidosis underwent transbronchial needle aspiration, endobronchial biopsy, and/or transbronchial lung biopsy. CTD-ILDs were diagnosed on clinical features, the detection of serum autoantibodies, and the presence of ILD on the chest CT. Idiopathic pulmonary fibrosis was mostly diagnosed on the presence of usual interstitial pneumonia pattern (definite or probable) on the chest CT. Hypersensitivity pneumonitis was diagnosed on a characteristic appearance on the chest CT and a definite history of exposure to offending antigens. In those with suspected IPF or HP, lung biopsy (mostly transbronchial lung cryobiopsy or surgical lung biopsy) was performed when the clinical or imaging findings were inconsistent. Wherever needed, the clinical, radiologic, and histopathologic data were reviewed by a multidisciplinary team comprising two or more pulmonologists, a radiologist, and a pathologist to assign a diagnosis. In general, patients were followed every 3–6 months. Information received on the death of any included patient was recorded.

### Incidence and prevalence of ILDs in our region

The crude annual incidence and prevalence of ILDs were calculated for the Tricity region. Our hospital is located in this region that comprises the three districts of Chandigarh (a Union Territory), Panchkula (in the state of Haryana), and Sahibzada Ajit Singh Nagar (in the state of Punjab). The estimated population of persons above the age of 12 years (henceforth, referred to as the ‘population’) of this region was obtained from the 2011 national census data [28]. Study participants residing in the Tricity and diagnosed during the study period were designated as ‘incident cases’. The crude annual incidence of ILDs per 100,000 population was calculated for each year (years 1–5) and the entire study duration (average annual incidence).

Next, the records of our clinic were searched for the reported deaths amongst Tricity residents. The study subjects or their next of kin were also contacted telephonically between March and April 2020 to obtain information on death or migration. Where the vital status of the subjects was unconfirmed, clinic records were searched for data on the radiologic features, lung function trends, the clinical condition at the last follow-up, and the visit pattern. Using this information, two authors (SD, RA) made informed assumptions on the vital status (alive or dead) of the subjects as of March 1, 2020. The point prevalence was then estimated on three different assumptions for defining the ‘prevalent cases’: (i) all the subjects with unavailable vital status were assumed dead; (ii) all of them were assumed alive; or (iii) the vital status was assigned using informed assumption. The proportion of each incident and prevalent ILD subtype was compared with another recent large (n = 1,084) study of ILDs in India (the ILD India registry) [18, 19].

Subsequently, all incident (and prevalent) cases were divided into eight age-and-gender groups using four age intervals (13–39 years, 40–59 years, 60–79 years, and ≥80 years). Direct standardization was performed against the 2011 national population [28]. The crude incidence and prevalence of the major ILD subtypes (sarcoidosis, IPF, CTD-ILD, HP, and others) were also calculated; standardization was avoided due to small samples.

### Calculation of burden of ILDs in India

Assuming the incidence and prevalence estimates for the Tricity to represent the entire country, the national incident and prevalent burden of ILD and its subtypes were calculated, based on the 2011 national population (primary estimates). To calculate the alternative estimates for the ILD subtypes, the average proportion of each ILD subtype from the current study and the ILD India Registry was multiplied by the overall national annual incident and prevalent burden of ILDs [18, 19]. Finally, estimates of all epidemiologic indices were calculated assuming different referral rates of ILD patients to our clinic (ranging between 10% and 90%, at intervals of 10%).

### Statistical analysis

Data were entered into worksheets using the computer program Microsoft Excel and analyzed using the statistical package SPSS version 22. Data are expressed as mean ± standard deviation (SD) or as number (percentage). Proportions were compared using the chi-squared test. A p-value of less than 0.05 was considered to reflect statistical significance.

## Results

We screened 2,042 subjects out of which 2,005 (mean [SD] age, 50.7 [13.6] years, 943 [47.0%] men; Table 1) were included; 37 subjects were excluded (26 refused to consent; 11 were finally diagnosed with other diseases [8 had tuberculosis, one each had a diffuse alveolar hemorrhage, pulmonary edema, and lymphangitis carcinomatosis]. Interventional procedures for tissue acquisition were performed in 966 (48.2%) subjects, of which 76.1% were diagnostic (Table 2). The diagnosis of the remaining subjects was made on clinico-radiologic information. Sarcoidosis was the most common (37.3%) major ILD subtype (Table 3), followed by CTD-ILDs (19.3%), IPF (17.0%), and HP (14.4%).

**Table 1:** Baseline characteristics of the subjects at study enrolment at the Chest Clinic (n = 2,005).

Characteristic	Sarcoidosis (n = 747)	CTD-ILD (n = 387)	IPF (n = 340)	HP (n = 288)	Others (n = 243)	All subjects (n = 2,005)
Age, years	44.4 ± 11.4	48.5 ± 11.9	65.8 ± 7.9	51.2 ± 13.6	51.5 ± 12.4	50.7 ± 13.6
Men	373 (49.9)	72 (18.6)	248 (72.9)	141 (49.0)	109 (44.9)	943 (47.0)
Body mass index (kg/m^2^)	26.4 ± 4.6	24.9 ± 4.7	24.2 ± 4.1	24.7 ± 5.3	25.7 ± 4.8	25.4 ± 4.7
Smokers	59 (7.9)	23 (5.9)	155 (45.6)	29 (10.1)	20 (8.2)	286 (14.3)
Spirometry	(n = 681)	(n = 364)	(n = 285)	(n = 255)	(n = 208)	(n = 1793)
Abnormality						
Normal	388 (57.0)	76 (20.9)	65 (22.8)	31 (12.2)	50 (24.0)	610 (34.0)
Obstructive	100 (14.7)	11 (3.0)	19 (6.7)	20 (7.8)	20 (9.6)	170 (9.5)
Restrictive	193 (28.3)	277 (76.1)	201 (70.5)	204 (80.0)	138 (66.3)	1013 (56.5)
Measurements (n = 1793)						
FVC	2.79 ± 0.91	1.82 ± 0.59	2.03 ± 0.69	1.77 ± 0.70	1.98 ± 0.74	2.23 ± 0.89
FVC %predicted	84.4 ± 18.1	66.4 ± 18.7	67.2 ± 18.2	58.1 ± 19.3	66.6 ± 19.1	72.2 ± 21.0
FEV_1_	2.19 ± 0.77	1.52 ± 0.47	1.65 ± 0.53	1.45 ± 0.56	1.60 ± 0.58	1.79 ± 0.70
FEV_1_%predicted	84.2 ± 20.7	72.4 ± 19.9	71.1 ± 18.2	61.7 ± 20.6	69.8 ± 19.9	74.8 ± 21.6
FEV_1_/FVC ratio	0.78 ± 0.09	0.84 ± 0.07	0.82 ± 0.09	0.83 ± 0.09	0.82 ± 0.09	0.81 ± 0.09

CTD-connective tissue disease, FEV_1_-forced expiratory volume in one second, FVC-forced vital capacity, HP-hypersensitivity pneumonitis, ILD-interstitial lung disease, IPF-idiopathic pulmonary fibrosis. All values are mean ± standard deviation or number with percentage.

**Table 2:** Details of invasive procedures performed during evaluation for obtaining the histological diagnoses of ILDs in study subjects (n = 966).

	Diagnostic	Contributory*	Non-diagnostic	Total number
Transbronchial lung biopsy	67 (41.9)	31 (19.4)	62 (38.8)	160
Any combination of transbronchial lung biopsy, endobronchial biopsy and transbronchial needle aspiration	575 (95.0)	2 (0.3)	28 (4.6)	605
Transbronchial lung cryobiopsy	47 (72.3)	8 (12.3)	10 (15.4)	65
Surgical lung biopsy	9 (90.0)	1 (10.0)	0	10
Bronchoalveolar lavage	4 (4.3)	18 (19.6)	70 (76.1)	92
Other diagnostic procedures†	34 (100)	0	0	34
Total	736 (76.2)	60 (6.2)	170 (17.6)	966

MDD- multidisciplinary discussion.

*Non-diagnostic but contributing important information to MDD

†Other diagnostic procedures included skin biopsy, liver biopsy, fine needle aspiration from lymph nodes, liver or spleen, and computed tomography guided lung biopsy

**Table 3:** Final diagnoses of study subjects assigned after complete evaluation in the Chest Clinic (n = 2,005).

Diagnosis	Number (percentage)
Sarcoidosis	747 (37.3)
Stage I	207 (10.3)
Stage II	372 (18.6)
Stage III	135 (6.7)
Stage IV	33 (1.6)
Connective tissue disease related ILD	387 (19.3)
Systemic sclerosis	146 (7.3)
Rheumatoid arthritis	102 (5.1)
Dermatomyositis/Anti-synthetase syndrome	18 (0.9)
Mixed connective tissue disease	15 (0.7)
Sjogren’s syndrome	10 (0.5)
Overlap syndrome	6 (0.3)
Systemic lupus erythematosus	5 (0.2)
Undifferentiated CTD	85 (4.2)
Idiopathic pulmonary fibrosis	340 (17.0)
Hypersensitivity pneumonitis	288 (14.4)
Others	243 (12.1)
Non-IPF idiopathic interstitial pneumonia	148 (7.4)
Nonspecific interstitial pneumonia	124 (6.2)
Cryptogenic Organizing Pneumonia	15 (0.7)
Respiratory Bronchiolitis- ILD/Desquamative Interstitial Pneumonia	7 (0.3)
Acute Interstitial Pneumonia	2 (0.1)
Occupational lung disease	24 (1.2)
Silicosis	17 (0.8)
Asbestosis	1 (0.1)
Arc welder’s lung	1 (0.1)
Pneumoconiosis, NOS	5 (0.2)
Drug-induced ILD	18 (0.9)
Unclassifiable	23 (1.1)
Miscellaneous	30 (1.5)
Pulmonary alveolar proteinosis	5 (0.2)
Chronic eosinophilic pneumonia	4 (0.2)
IgG4 associated ILD	4 (0.2)
ANCA-associated ILD	3 (0.1)
Pulmonary Langerhans cell histiocytosis	2 (0.1)
Cystic lung disease, NOS	2 (0.1)
Pulmonary alveolar microlithiasis	2 (0.1)
Lymphangioleiomyomatosis	2 (0.1)
Idiopathic pulmonary hemosiderosis	2 (0.1)
Psoriasis-related ILD	2 (0.1)
CVID associated LIP	1 (0.1)
Talcosis	1 (0.1)

ANCA-antineutrophil cytoplasmic antibody, CTD-connective tissue disease, CVID-common variable immunodeficiency, Ig-Immunoglobulin, ILD-interstitial lung disease, IPF-Idiopathic pulmonary fibrosis, LIP-lymphocytic interstitial pneumonia, NOS-not otherwise specified.

Of the 517 subjects residing in the Tricity region (Table 4), 409 were incident cases. Amongst incident cases, the proportions of all ILD subtypes except CTD-ILD were different between the current study and the ILD India registry. Among prevalent cases, the proportions of sarcoidosis and HP were different between the two studies (Table 4). The Tricity region’s population for individuals >12 years of age was 2,028,557. Accordingly, the crude annual incidence of ILDs per 100,000 population for the five successive years of our study period was 4.29, 3.94, 3.89, 4.63, and 3.40, respectively, yielding an average of 4.03 (Table 5). For the age groups 13–39, 40–59, 60–79, and ≥80 years, the respective estimates for annual incidence (per 100,000) for men were 1.39, 5.11, 13.13, and 7.94, respectively, while for women, these were 1.58, 9.06, 14.13, and 1.67, respectively.

**Table 4:** Comparison of the spectrum of interstitial lung diseases amongst incident and prevalent cases and year-wise distribution of incident cases in the Tricity region.

**ILD subtype**	**All cases**	**Incident cases**	**ILD India registry study**	**P value**	
Sarcoidosis	209 (40.4)	159 (38.9)	85 (7.8)	<0.001	
CTD-ILD	85 (16.4)	70 (17.1)	151 (13.9)	0.14	
IPF	111 (21.5)	87 (21.3)	148 (13.7)	<0.001	
HP	62 (12.0)	58 (14.2)	513 (47.3)	<0.001	
Others	50 (9.7)	35 (8.6)	187 (17.3)	<0.001	
Total	517	409*	1084		
	**Prevalent cases** ^**a**^	**Prevalent cases** ^**b**^	**Prevalent cases** ^**c**^	**ILD India registry study**	**P value**
Sarcoidosis	199 (48.3)	190 (50.0)	196 (49.2)	38 (9.5)	<0.001
CTD-ILD	71 (17.2)	63 (16.6)	69 (17.3)	80 (20.1)	0.37
IPF	52 (12.6)	46 (12.1)	47 (11.8)	37 (9.3)	0.29
HP	52 (12.6)	47 (12.4)	50 (12.6)	190 (47.6)	<0.001
Others	38 (9.2)	34 (8.9)	36 (9.0)	54 (13.5)	0.06
Total	412	380	398	399	

CTD-connective tissue disease, HP-hypersensitivity pneumonitis, ILD-interstitial lung disease, IPF-idiopathic pulmonary fibrosis.

*The number of incident cases in year 1, 2, 3, 4, and 5 were 87, 80, 79, 94, and 69, respectively. All values represent number (percentage) Prevalent cases calculated according to different assumptions for subjects with unknown vital status on March 1, 2020: All assumed to be alive^a^, all assumed to be dead^b^, or status assigned by best assumptions on the vital status by two authors^c^.

The p values are derived by applying the chi-squared test for the difference in proportions for each ILD subtype between the current study and the ILD India registry study for incidence and prevalence (by the best assumptions method).

**Table 5:** Incidence and prevalence of interstitial lung diseases in the Tricity region and the estimated national incident and prevalent burden according to different assumptions of referral rates.

Incidence	Calculated	Referral rate								
(per 100,000 population)		0.10	**0.20**	**0.30**	**0.40**	0.50	0.60	0.70	0.80	0.90
Year 1	4.29	42.9	**21.4**	**14.3**	**10.7**	8.6	7.1	6.1	5.4	4.8
Year 2	3.94	39.4	**19.7**	**13.1**	**9.9**	7.9	6.6	5.6	4.9	4.4
Year 3	3.89	38.9	**19.5**	**13.0**	**9.7**	7.8	6.5	5.6	4.9	4.3
Year 4	4.63	46.3	**23.2**	**15.4**	**11.6**	9.3	7.7	6.6	5.8	5.1
Year 5	3.40	34.0	**17.0**	**11.3**	**8.5**	6.8	5.7	4.9	4.3	3.8
Mean annual incidence (crude)	4.03	40.3	**20.2**	**13.4**	**10.1**	8.1	6.7	5.8	5.0	4.5
Mean annual incidence (standardized)	4.19	41.9	**21.0**	**14.0**	**10.5**	8.4	7.0	6.0	5.2	4.7
National annual incidence (crude)	35625	356248	**178124**	**118749**	**89062**	71250	59375	50893	44531	39583
National annual incidence (standardized)	37059	370586	**185293**	**123529**	**92646**	74117	61764	52941	46323	41176
Prevalence	Calculated	Referral rate								
(per 100,000 population)		0.10	**0.20**	**0.30**	**0.40**	0.50	0.60	0.70	0.80	0.90
Crude Prevalence (1)^a^	20.31	203.1	**101.6**	**67.7**	**50.8**	40.6	33.9	29.0	25.4	22.6
Crude Prevalence (2)^b^	18.73	187.3	**93.7**	**62.4**	**46.8**	37.5	31.2	26.8	23.4	20.8
Crude Prevalence (3)^c^	19.62	196.2	**98.1**	**65.4**	**49.0**	39.2	32.7	28.0	24.5	21.8
Prevalence^c^ (standardized)	20.29	202.9	**101.4**	**67.6**	**50.7**	40.6	33.8	29.0	25.4	22.5
National burden^c^ (crude)	173334	1733336	**866668**	**577779**	**433334**	346667	288889	247619	216667	192593
National burden^c^ (standardized)	179224	1792240	**896120**	**597413**	**448060**	358448	298707	256034	224030	199138

Prevalence calculated according to different assumptions for subjects with unknown vital status on March 1, 2020: All assumed to be alive^a^, all assumed to be dead^b^, or status assigned by best assumptions on the vital status^c^. The values in bold font provide the range based on our best assumptions of the referral rates.

A total of 380 Tricity subjects were alive, 100 had died, five had migrated, while the vital status remained unknown for 32, as on March 1, 2020. The total number of prevalent cases of ILDs in the region were 412, 380, and 398 based on whether the 32 subjects with unknown vital status were assumed to be alive, dead, or assigned a status using the best assumptions, respectively. The crude prevalence of ILDs in the region according to the ‘best assumptions on vital status’ method was 19.62 cases per 100,000 population. Assuming 20–40% referral rates to our center, the estimated crude annual incidence and prevalence were 10.1–20.2 and 49.0–98.1, respectively, per 100,0000 population (Table 5). Accordingly, the estimated standardized national annual incident cases of ILDs ranged between 92,646 to 185,293 cases, while the national (prevalent) burden was estimated at 448,060 to 896,120.

Assuming 20–40% referral, the estimated crude annual incidence rates (per 100,000 population) for sarcoidosis, CTD-ILDs, IPF, HP, and other ILDs were 3.9–7.8, 1.7–3.5, 2.1–4.3, 1.4–2.9, and 0.9–1.7, respectively (Table 6). The respective estimates for the prevalence (per 100,000 population) were 24.2–48.3, 8.5–17.0, 5.8–11.6, 6.2–12.3, and 4.4–8.9. The best primary estimates for the crude national burden of all ILDs, sarcoidosis, CTD-ILD, IPF, HP, and other ILDs (in thousands) were 433–867, 213–427, 75–150, 51–102, 54–109, and 39–78 (Table 6). The respective alternative estimates (in thousands) were: sarcoidosis, 127–254; CTD-ILD, 81–162; IPF, 46–91; HP, 130–261; other ILDs, 49–98.

**Table 6:** Incidence and prevalence of various subtypes of interstitial lung diseases in the Tricity region and estimated national burden according to different assumptions of referral rates to our center.

ILD subtype		Referral rate								
		0.1	0.2	0.3	0.4	0.5	0.6	0.7	0.8	0.9
Sarcoidosis										
Incidence	1.57	15.7	**7.8**	**5.2**	**3.9**	3.1	2.6	2.2	2.0	1.7
Prevalence	9.66	96.6	**48.3**	**32.2**	**24.2**	19.3	16.1	13.8	12.1	10.7
National incident burden	13849	138493	**69246**	**46164**	**34623**	27699	23082	19785	17312	15388
National burden	85360	853603	**426801**	**284534**	**213401**	170721	142267	121943	106700	94845
Alt national incident burden	5822	58221	**29111**	**19407**	**14555**	11644	9704	8317	7278	6469
Alt national burden	50891	508909	**254455**	**169636**	**127227**	101782	84818	72701	63614	56545
CTD-ILD										
Incidence	0.69	6.9	**3.5**	**2.3**	**1.7**	1.4	1.2	1.0	0.9	0.8
Prevalence	3.40	34.0	**17.0**	**11.3**	**8.5**	6.8	5.7	4.9	4.3	3.8
National incident burden	6097	60972	**30486**	**20324**	**15243**	12194	10162	8710	7621	6775
National burden	30050	300503	**150251**	**100168**	**75126**	60101	50084	42929	37563	33389
Alt national incident burden	5273	52733	**26367**	**17578**	**13183**	10547	8789	7533	6592	5859
Alt national burden	32405	324049	**162024**	**108016**	**81012**	64810	54008	46293	40506	36005
IPF										
Incidence	0.86	8.6	**4.3**	**2.9**	**2.1**	1.7	1.4	1.2	1.1	1.0
Prevalence	2.32	23.2	**11.6**	**7.7**	**5.8**	4.6	3.9	3.3	2.9	2.6
National incident burden	7578	75779	**37890**	**25260**	**18945**	15156	12630	10826	9472	8420
National burden	20469	204690	**102345**	**68230**	**51173**	40938	34115	29241	25586	22743
Alt national incident burden	5607	56074	**28037**	**18691**	**14018**	11215	9346	8011	7009	6230
Alt national burden	18269	182685	**91343**	**60895**	**45671**	36537	30448	26098	22836	20298
HP										
Incidence	0.57	5.7	**2.9**	**1.9**	**1.4**	1.1	1.0	0.8	0.7	0.6
Prevalence	2.46	24.6	**12.3**	**8.2**	**6.2**	4.9	4.1	3.5	3.1	2.7
National incident burden	5052	50519	**25260**	**16840**	**12630**	10104	8420	7217	6315	5613
National burden	21776	217756	**108878**	**72585**	**54439**	43551	36293	31108	27219	24195
Alt national incident burden	13625	136248	**68124**	**45416**	**34062**	27250	22708	19464	17031	15139
Alt national burden	52196	521958	**260979**	**173986**	**130490**	104392	86993	74565	65245	57995
Other ILDs										
Incidence	0.35	3.5	**1.7**	**1.2**	**0.9**	0.7	0.6	0.5	0.4	0.4
Prevalence	1.77	17.7	**8.9**	**5.9**	**4.4**	3.5	3.0	2.5	2.2	2.0
National incident burden	3049	30486	**15243**	**10162**	**7621**	6097	5081	4355	3811	3387
National burden	15678	156784	**78392**	**52261**	**39196**	31357	26131	22398	19598	17420
Alt national incident burden	5297	52972	**26486**	**17657**	**13243**	10594	8829	7567	6621	5886
Alt national burden	19573	195734	**97867**	**65245**	**48934**	39147	32622	27962	24467	21748

Alt-alternative estimates of, CTD-connective tissue disease, HP-hypersensitivity pneumonitis, ILD-interstitial lung disease, IPF-idiopathic pulmonary fibrosis.

All values for prevalence are per 100,000 and those for incidence are per 100,000 population per year. The prevalence was calculated based on best assumptions on the vital status for subjects with unknown status on March 1, 2020. The alternative estimates were prepared by averaging the proportion of each ILD subtype from the current study and the ILD India Registry study. The values in bold font provide the range based on our best assumptions of the referral rates.

## Discussion

The estimated crude annual ILD incidence and prevalence in our region (per 100,000 population) were 10.1–20.2, and 49.0–98.1, respectively, while the standardized national prevalent burden was 0.45–0.89 million. To our knowledge, this is the first study on the incidence, prevalence, and burden of ILDs from a developing country. It is also the largest single-center experience of the spectrum of ILDs diagnosed using contemporary guidelines.

Our primary estimates were derived from prospectively collected data in a hospital-based registry. Our hospital is the largest referral center in the region north of the national capital offering specialized care for sarcoidosis and other ILDs. Yet, it is expected that not all patients in this region would have registered with us. In a survey, it was found that about 80% of the primary physicians in our region referred suspected patients with IPF to higher centers [29]. This region has two other major public sector hospitals, five large private hospitals, and several independent private clinics providing care to ILD patients. Other potential factors hampering enrolment into our registry are misdiagnosis at the primary level (such as sarcoidosis and HP wrongly diagnosed as tuberculosis, and IPF as chronic obstructive pulmonary disease), patient hesitancy to seek tertiary care, and patients with sarcoidosis and CTD-ILD being treated by rheumatologists and internists. Therefore, we estimated tentatively that about 20–40% of the ILD patients from the region got registered at our clinic. The alternative estimates for the ILD subtypes derive from a larger dataset including the current study and a large multicenter study of 1,084 subjects from different regions of the country, and thus may be more representative [18].

Our best estimated crude annual ILD incidence (10.1–20.2/100,000) lies within the overall range (1–70.1 per 100,000 population) reported in other studies (Table 7). It is close to that reported in one of the most well-performed studies of ILD epidemiology in recent times from Greater Paris, France (19.4/100,000) [13]. To our knowledge, ILD prevalence has been reported by only four previous studies and ranges from 6.3–97.9 per 100,000 population [4, 5, 10, 13]; our estimates (49.0–98.1/100,000) fall on the higher side of this range (Table 7).

**Table 7:** Incidence of interstitial lung diseases found in previous studies.

Author (Year)	Country	Population	Annual Incidence	Prevalence
Coultas, et al. (1994) [4]	United States	480,577	31.5 (males)	80.9 (males)
			26.1 (females)	67.2 (females)
Thomeer, et al. (2001) [5]	Belgium	5,768,925	1.0	6.27
Lopez-Campos, et al. (2004) [6]	Spain	6,848,243	3.6	
Xaubet, et al. (2004) [7]	Spain	6,700,000	7.6	
Tinelli, et al. (2005) [8]	Italy	450,000	2.9	
Kornum, et al. (2008) [9]	Denmark	5,400,000	42.7 (crude)	
			31.3 (standardized)	
Karakatsani, et al. (2009) [10]	Greece	5,600,000	4.6	17.3
Hyldgaard, et al. (2014) [11]	Denmark	1,200,000	4.1	
Musellim, et al. (2014) [12]	Turkey	-	25.8	
Duchemann, et al. (2017) [13]	France	1,194,601	19.4	97.9
Storme, et al. (2017) [14]	Canada	17,956	32 (crude)	
			80 (standardized)	
Choi, et al. (2018) [15]	Republicof Korea	312,529	70.1	
Hilberg, et al. (2018) [16]	Denmark	5,500,000	17.6	
Present study	India	2,008,611	10.1–20.2 (crude)	49.0–98.1 (crude)
			10.5–21.0 (standardized)	50.7–101.4 (standardized)

The annual incidence and prevalence represent crude estimates, unless otherwise specified.

The standardized annual ILD incidence in the current study (10.5–21.0/100,000) is about 10–20 times lower than that for tuberculosis in India (199/100,000) [30]. Moreover, the national burden of ILDs (0.45–0.89 million) is about 90 times lower than that of chronic obstructive pulmonary disease (55.3 million) and about 60 times lower than that of asthma (37.9 million) [31]. Even for allergic bronchopulmonary aspergillosis, a less common respiratory disorder, the best estimated total national burden is 0.86–1.52 million, about twice that of ILDs [32]. With a population prevalence of less than 10/10,000, the ILDs even as a single group remain rare disorders [33]. However, the total of 0.45–0.89 million cases represents a significant disease burden at the national level. The alternative estimates suggest that sarcoidosis (127.2–254.5 thousand cases) and HP (130.5–260.9 thousand cases) have a particularly significant presence in the country. The remarkable burden of ILDs estimated in this study might sensitize government and non-government healthcare agencies towards greater resource allocation for these diseases.

The annual incidence (3.9–7.8) and prevalence (24.2–48.3) of sarcoidosis (per 100,000 population) in the present study are like those reported from France (incidence, 4.9; prevalence, 30.2) and lie within the overall range (incidence, 0.13–17.8; prevalence, 2–160) reported previously [13, 34]. Our annual incidence (2.1–4.3) and prevalence (5.8–11.6) of IPF (per 100,000 population) are also like those in France (incidence, 2.8; prevalence, 8.2), less than Italy and Canada, but higher than Belgium and Greece [5, 10, 13, 35, 36]. The presence of CTD-ILD in our population (incidence, 1.7–3.5; prevalence, 8.5–17.0 per 100,000 population) is higher than the previously reported range (incidence, 0.07–3.3; prevalence, 0.47–12.1), owing to either an actual difference in occurrence or higher referral rates [34]. Our IPF incidence is higher but prevalence lower than CTD-ILD reflecting the shorter survival in IPF [37]. More importantly, HP is much more frequent in our population (incidence, 1.4–2.9; prevalence, 6.2–12.3) than in developed countries including Belgium (incidence, 0.12; prevalence, 0.81), France (incidence, 0.9; prevalence, 2.3), and the United States (incidence, 1.28–1.94; prevalence, 1.67–2.71) per 100,000 population, as suggested previously by Singh et al [5, 13, 18, 38]. For HP, the alternative estimates of the national burden found by averaging the proportion in the current and the ILD India registry study are even higher (more than two times) than those from our study alone (Table 6) [19]. The alternative estimates suggest that sarcoidosis and HP have an almost equal prevalent burden contrary to other world regions, where sarcoidosis and IPF are the most prevalent ILDs [4, 13, 36].

**Fig 1:**
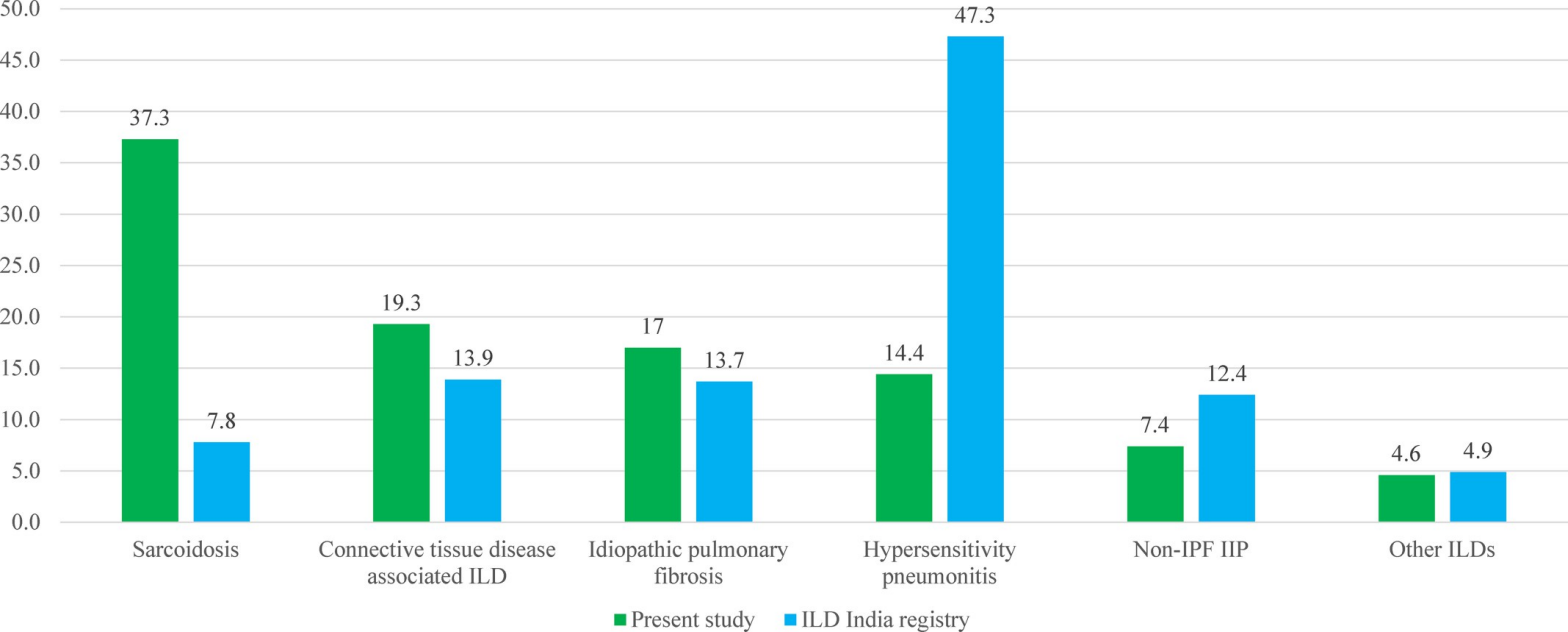
Comparison of spectrum of interstitial lung diseases in this study and a large (n = 1,084) multicenter study from India. [18] The numbers represent percentage of subjects diagnosed with the condition.

The ILD spectrum in India remains contentious. In our previous study, sarcoidosis (42.2%) was the commonest ILD (n = 803), followed by IPF (21.2%), CTD-ILD (12.7%), and HP (10.7%). The present analysis, which includes the patient population of our previous study, reveals a slightly different spectrum. Though sarcoidosis (37.3%) remains the commonest, the second most common ILD is CTD-ILD (19.3%) instead of IPF, which is placed third now (17.0%). The proportion of HP is slightly higher at 14.4%. These differences might result from changes in referral practices, better awareness, and improved use of various diagnostic techniques. The current spectrum still differs from the ILD India registry, where HP was the most common ILD subtype (Fig 1) [18].

This study has a few limitations. The estimates draw on several assumptions including the vital status of subjects with missing follow-up data, referral rates, and uniform ILD incidence across the country. We used the 2011 national census data as the most recent available resource for the population estimates. This might be inaccurate for our study period owing to population growth. Therefore, we have provided broad estimate ranges considering different referral rates and presented alternative estimates to account for the different ILD spectrum in the ILD India registry. Our estimates are thus crude and tentative approximations like the ‘Fermi estimates’ [32, 39]. Such estimates provide rough assessments, that can vary by a one-log precision. Even rough estimates are potentially valuable as they may guide future investigations, especially community-based studies. Our study’s strength is that ILD diagnosis was made at a referral center by an experienced team following the latest diagnostic standards.

In conclusion, the overall incidence and prevalence of ILDs in India are like those found in the developed world. However, sarcoidosis and HP have the highest prevalent burden according to the alternative estimates, contrary to the findings from developed countries. Despite being rare, the ILDs represent a significant disease burden. Population-based, multicenter studies from different geographic regions are required to better define the epidemiology of ILDs in India.
